# Prevalence of Childhood Atopic Dermatitis: An Urban and Rural Community-Based Study in Shanghai, China

**DOI:** 10.1371/journal.pone.0036174

**Published:** 2012-05-01

**Authors:** Feng Xu, Shuxian Yan, Fei Li, Minqiang Cai, Weihan Chai, Minmin Wu, Chaowei Fu, Zhuohui Zhao, Haidong Kan, Kefei Kang, Jinhua Xu

**Affiliations:** 1 Department of Dermatology, Huashan Hospital, Shanghai Medical College, Fudan University, Shanghai, People's Republic of China; 2 Xinjing Community Health Service Center, Shanghai, People's Republic of China; 3 Department of Dermatology, Jiading District Traditional Chinese Medicine Hospital, Shanghai, People's Republic of China; 4 School of Public Health, Key Lab of Public Health Safety of the Ministry of Education, Shanghai Medical College, Fudan University, Shanghai, People's Republic of China; University of Montreal, Canada

## Abstract

**Background:**

Atopic dermatitis (AD) is a common inflammatory and chronically relapsing disorder with increasing prevalence. However, little is known about its prevalence in Shanghai, the top metropolitan of China. This study will estimate and compare the prevalence of AD in urban and rural areas in representative samples of 3 to 6-year-old children in Shanghai.

**Methodology/Principal Findings:**

A descriptive cross-sectional study was performed. Pre-school children were obtained by cluster sampling from 8 communities in different districts in Shanghai. The main instrument was the core questionnaire module for AD used in the U.K. Working Party's study. All the data were statistically analyzed by EpiData 3.1 and SPSS16.0. A total of 10436 children completed the study satisfactorily, with a response rate of 95.8%. The prevalence of AD in 3 to 6-year-old children was 8.3% (Male: 8.5%, Female: 8.2%). The prevalence in urban areas of Shanghai was gradiently and significantly higher than that in rural areas. The highest prevalence was in the core urban area (10.2% in Xuhui Tianping) vs. the lowest far from the urban areas (4.6% in Chongming Baozhen).

**Conclusions/Significance:**

The prevalence of AD was 8.3% (95%CI: 7.6%–9.1%) in children aged 3 to 6 in Shanghai. The prevalence of AD decreased from the center to the rural areas in Shanghai.

## Introduction

Atopic dermatitis (AD) is a common inflammatory disease characterized by intense itch and eczematous lesions, which has become an important health problem in children worldwide. There are considerable differences in prevalence between and within countries due to geographic distributions and economical development. A high prevalence of AD is noted in countries such as Sweden, Japan, New Zealand, United Kingdom, Portugal and Thailand, opposed to the countries with lower prevalence rates[1] such as in Iran, Albania, India, Singapore and Spain. In China. A higher prevalence was found in Beijing and Shanghai, reported by Gu Heng in 2002, while a lower prevalence was found in Shenyang[2]. Since then, with the rapid economic development in Shanghai, there has been no follow-up study of the prevalence of AD in children of this city. The objective of this work was to evaluate and compare the prevalence of AD in children aged 3 to 6 year-old in Shanghai with special attention to the difference between urban and rural areas.

## Methods

### Selection of population

**Figure 1:**
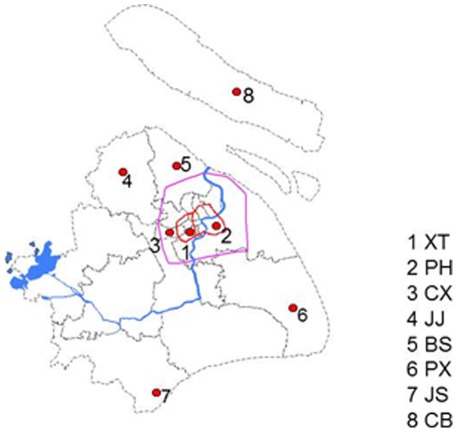
Location of eight communities. The urban area of Shanghai is encircled as the Inner Ring (red) and urbanized suburb of Shanghai is encircled as the Outer Ring (pink).

The survey was conducted in eight communities, which are listed in Figure. 1 from March to July, 2010. According to the geographic distribution in Shanghai, eight communities were randomly selected from different areas. The selection of communities was stratified with 2 from urban area, 2 from suburban area and 4 from rural area. All the kindergardens registered in the communities selected were involved in this study by cluster sampling. Children enrolled in the kindergartens in these eight communities were all surveyed in this study. 1-Xuhui Tianping (XT) and 2-Pudong Huamu (PH) are in the center of Shanghai. 3-Changning Xinjing (CX) is at the boundary of the center area, about 10 kilometers from the city center. 4-Jiading Juyuan(JJ)and 5-Baoshan Sitang(BS) are about 30 to 40 kilometers from the center of Shanghai and have experienced significant economic development in the past decade. 6-Nanhui Xinchang(NX), 7-Jinshan Sanyang (JS) and 8-Chongming Baozheng (CB) are typical of the countryside around Shanghai, about 60 to100 kilometers from the center of Shanghai, and much farther away than 4 and 5.

### Data collection and diagnostic criteria

One teacher in each kindergarden was responsible for the interview (including communicate with the parents and explain the questions if necessary). All these teachers in charge were undertaken the uniform training by the professional dermatologist to ensure they fully understood the questions in the questionnaire before they helped the children's parents fill out the questionnaire. Personal basic information, such as date of birth, sex, address, telephone number, etc. was recorded and questions based on the U.K. Working Party's diagnostic criteria (UK criteria) for AD were used. In our research, the questionnaire was translated into local language by the teachers in the kindergarten if the parents couldn't understand the written language. The study and the questionnaire were approved by the Ethics Committee of Huashan Hospital, Fudan University, Shanghai, China.

The diagnostic UK criteria [3] require: itchy skin (or parental report of scratching or rubbing in a child) plus 3 or more of the following:

1. History of involvement of the skin creases such as folds of elbows, behind the knees, front of ankles or around the neck (including cheeks in those under 10 years old).
2. A personal history of asthma or hay fever (or history of atopic disease in a first degree relative in those under 4 years old).
3. A history of generally dry skin in the last year.
4. Visible flexural dermatitis (or dermatitis involving the cheeks/forehead and outer aspect of the limbs in children under 4).
5. Onset under the age of 2.

If the child had a history of pruritus and 3 or more minor criteria, he/she would be diagnosed as AD.

We selected inclusion criteria of 3 to 6 years old as the majority of AD patients have early childhood onset, and this also provides many years of life for sequential follow-up studies.

### Data analysis

Data were entered on the database (Epidata 3.1). A χ2 test was used to calculate the difference between groups. OR, 95% confidence interval (CI), and significance values for the prevalence of AD were calculated using the SPSS statistics analysis package (SPSS for Windows, version 16.0, Chicago, IL, USA). A p-value <0.05 was considered statistically significant. Spearman bivariate analysis was used to evaluate the correlation between the prevalence of AD and GDP (Gross Domestic Product) per capita. Correlation was significant at the 0.01 level.

## Results

Of the 10891 subjects, a total of 10436 children were completely surveyed. The responding rate was 95.8%. The reason for no response were sickness or travel of children that their parents could not be informed. But these missing data didn't influence the final results.

A total of 5383 boys and 5053 girls participated in the study and the average age was 5.11±0.90 years old. The prevalence of AD based on the questionnaire was 8.3% (95%CI: 7.6%–9.1%). Boys with AD (8.5% [95%CI: 7.0%–9.9%]) slightly prevailed over girls (8.2% [95%CI:7.3%–9.1%]); this difference was not statistically significant(P value>0.05). The prevalence of AD in boys but not girls decreased from 3 to 5 years old. Table 1 shows the prevalence of AD by ages and gender.

**Table 1:** Atopic Dermatitis age-grouped by gender.

age	male			female			Total
	population	AD	%	population	AD	%	%
3∼	1152	110	9.5%	1042	86	8.3%	8.9%
4∼	1729	149	8.6%	1606	122	7.6%	8.1%
5∼	1854	140	7.6%	1797	150	8.3%	7.9%
6∼	648	56	8.6%	608	57	9.4%	9.0%

The questionnaire was used based on UKWP criteria for AD diagnosis.

**Figure 2:**
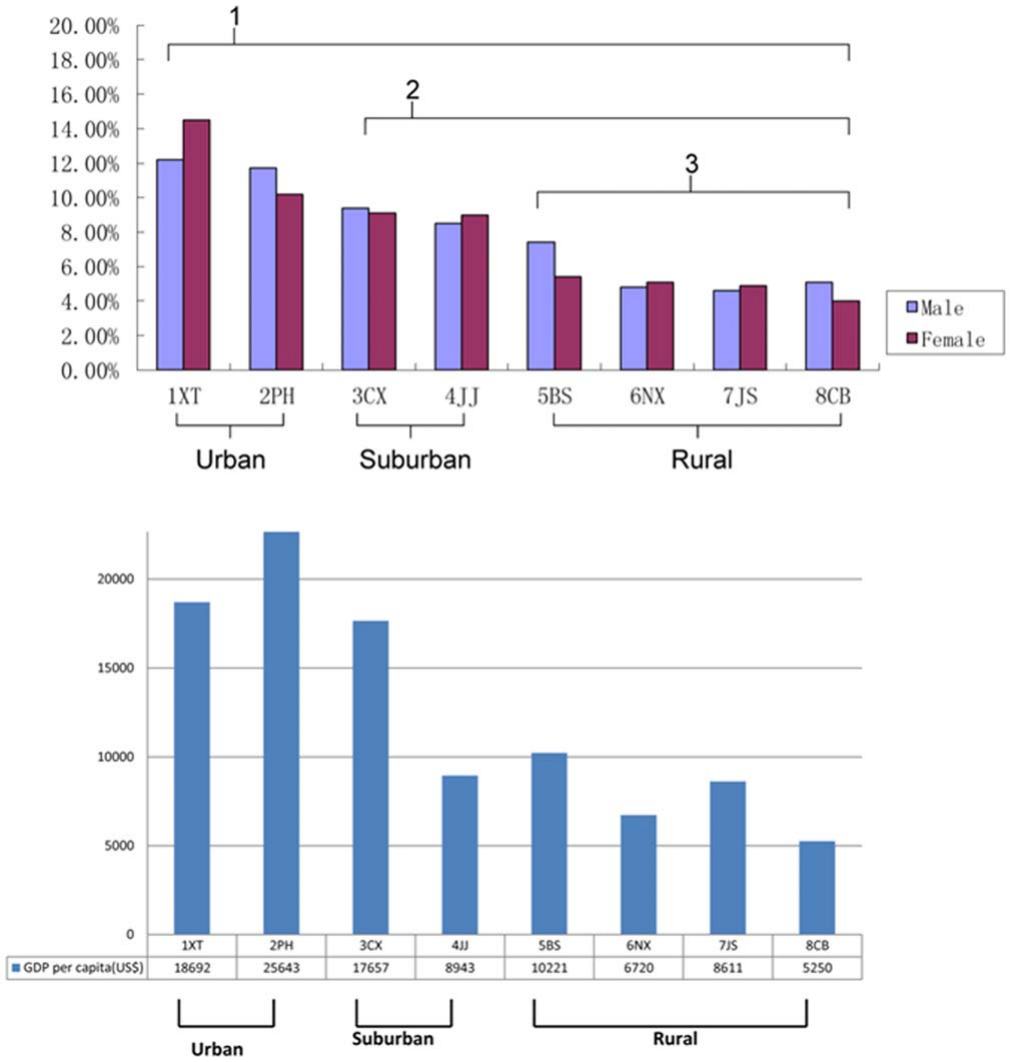
Prevalence of AD in different districts; GDP per capita in different districts. Figure 2a Prevalence of AD in different districts. 1 The prevalence of Urban (#1XT and #2PH) showed statistically significant difference to that of other districts (P<0.05). 2 The prevalence of Suburban (#3CX and #4JJ) showed statistically significant difference to that of the rural areas (P<0.05). 3. Meanwhile the prevalence of different communities in each unit (Urban, Suburban and Rural) showed no statistically significant difference (P>0.05). Figure 2b GDP per capita in different districts. The prevalence of AD has significant correlation with GDP per capita in different districts.(rs = 0.786, P = 0.001)

The prevalence of AD according to geographic distribution (Figure. 1 and Figure. 2a) was obviously higher in the core area of the city. The highest prevalence of AD was in 1.XT community, which was located in the center of Shanghai and the lowest prevalence was in 8-CB community, which was typical of the countryside around Shanghai and farthest away from the center of Shanghai (OR = 1.509, 95% CI 1.111–2.050). Figure. 2b shows GDP per capita of different districts in 2008. Table 2 shows the detailed data of the prevalence of AD in eight different communities. The prevalence of AD has significant correlation with GDP per capita in different districts (r_s_ = 0.786, P = 0.001).

**Table 2:** Atopic Dermatitis in 3 to 6 years old children populated in the different communities.

Community	population	AD		Prevalence		P value*
			Male	Female	Total	
1XT	1214	162	12.2%	14.5%	13.3%	
2PH	1642	180	11.7%	10.2%	11.0%	>0.05
3CX	2309	214	9.4%	9.1%	9.3%	<0.05
4JJ	958	84	8.5%	9.0%	8.8%	<0.05
5BS	1503	96	7.4%	5.4%	6.4%	<0.05
6NX	987	49	4.8%	5.1%	5.0%	<0.05
7JS	990	47	4.6%	4.9%	4.7%	<0.05
8CB	833	38	5.1%	4.0%	4.6%	<0.05

The prevalence of AD ordered by the distance from the core urban to the rural communities surveyed in Shanghai.

* P value refers to the prevalence of each community compared with the prevalence of 1XT.

## Discussion

In this large sample of community-based cross-sectional study, we enrolled 10436 pre-school children aged 3 to 6 in Shanghai using UK criteria. The prevalence of AD was 8.3% (95%CI: 7.6%–9.1%). A high response rate of 95.8% (n = 10436/10891) with statistical results by UK criteria have made this updated data reliable [2]. The symptoms of AD as a rule are expressed within the first two years of life, thus, this data provides a full span of life time for later sequential follow-up studies.

There has been no study reporting the prevalence of AD in Shanghai since 2002. Currently, the prevalence in this study (8.3%) was much higher relative to the 2.78% derived from Gu's study in Shanghai and 4.75% in Beijing in 2002 using the same diagnostic criteria [4]. We then considered the economic development in terms of GDP; the data showed that GDP per capita in Shanghai has tremendously grown from US$4900 in 2002 to over US$10,000 in 2010. We found, interestingly, that the AD prevalence in the center of Shanghai was much higher than that of rural area (10.2% in 1XT vs 4.6% in 10CB, P<0.05). The odds ratio was 1.509. The prevalence of AD has significant correlation with GDP per capita in different districts (r_s_ = 0.786, P = 0.001). Some suburban areas, such as 4JJ, have a relatively higher prevalence than that of the other rural areas (5BS). Schram et al [5] had analyzed all twenty-six primary studies comparing the prevalence of eczema between urban and rural populations. Nineteen showed a higher risk for eczema in an urbanized area, of which 11 were significant. Our community-based study now adds more data supporting a higher risk for AD in urban areas. Although China is a developing country, Shanghai is currently a renowned metropolitan city, especially in the core of the city such as the areas located at #1 and #2 shown in Fig. 1. Moreover, in the past 10 years, in addition to the #1 and #2 areas, some areas have also been developing rapidly such as #4JJ and #5BS, and become urbanized with the high-speed development paralleled with #1 and #2, which may account for the increment of the prevalence of AD in Shanghai. In fact, in recent years, industrial manufacturing has grown rapidly in #4JJ and #5BS, in contrast, #8(CB) still keeps in old production way as some other communities in the countryside. Indeed, there are several variables among rural and urban areas, which might contribute to the differences found in prevalence in Shanghai. Pollution is usually higher in urban areas, as the traffic in urban areas is notably higher than in rural areas. Dotterud et al. [6] found a significant relative risk (3.0, 95%CI 2.5–3.5) for prevalence of eczema in polluted versus nonpolluted areas. Other possible factors that might contribute to the effects are exposure to animals, maternal age, overcrowding in an apartment, differences in food (e.g. processed vs. fresh), socioeconomic factors and time spent indoors[7]. Further studies on environmental circumstances are currently ongoing in our group to reveal the factors associated with a higher prevalence of AD in urban areas.

The result of our study was in agreement with the results of the previous studies performed in other Asian areas and countries nearby. Taiwan found an overall prevalence of 6.7% of AD among the whole population and 9.6% among the people less than 20 years old reported in 2010[8]. In the study of ISAAC (International Study of Asthma and Allergy in Childhood), the overall prevalence of AD in Japan was 11.2% (11.8% among 6–7 years old and 10.5%11–12 years old [9].

According to the latest data reported by Tatyana E. Shaw, prevalence ranged from 8.7 to 18.1% among different areas in the United States with the highest prevalence in many districts of the East Coast states. Metropolitan living style and educational level were found to be associated with prevalence of AD [10].

It is obvious that the economic condition in Shanghai has been rapidly developing and is now similar to that in those areas or countries. The prevalence of AD in Shanghai has become as high as that of other developed areas in Asia. Meanwhile, with comparison of European and American areas, the prevalence of AD is still lower. According to the ISAAC's reports, the prevalence of AD surveyed in several European countries ranged from 13.5% to 21.4% [11]–[14]. Most likely, differences in diet and lifestyles, environmental and educational levels may add to the different prevalence between eastern and western countries.

Our study gives a strong evidence of the representation and large samples of children from different districts, including urban, urbanized suburb and rural areas and high response rate. Before the survey, kindergarten staffs were trained to explain the questionnaire and to help the parents ascertain flexural dermatitis. AD is usually the first symptom of the “atopic march”, so we studied children 3 to 6 years old to allow investigation of the entire march in the following years. While there is still some limitations that this was a questionnaire study without examination of each case by a dermatologist. Misinterpretation could still occur in different populations, and recall bias may exist.
